# Insights from an awareness survey on Oral Submucous Fibrosis (OSMF)

**DOI:** 10.6026/97320630016999

**Published:** 2020-12-31

**Authors:** Harippriya Karthikeyan, Abilasha Ramasubramanian, Visalakshi Ramanathan

**Affiliations:** 1 Saveetha Dental College, Saveetha Institute of Medical and Technical Sciences, Saveetha University, Chennai 77, India

**Keywords:** Oral Submucous Fibrosis, patients, causes, treatment modalities, awareness

## Abstract

Oral Submucous Fibrosis (OSMF) is characterized by Juxta-epithelial inflammation with oedema, fibroblasts, neutrophil and eosinophil inflammatory infiltrate. Therefore, it is of interest to gain insights from an awareness survey on ORAL SUBMUCOUS FIBROSIS (OSMF). The sample size was 100 patients who had participated in this survey in Tamilnadu, India where 93% of the subjects used tobacco products. 81% of the patients were not aware of the malignancy caused by OSMF. A significant number of males use more tobacco than females (p value = 0.006 (<0.05) in the sample dataset. Thus, there is a need to create awareness on OSMF among the general population to combat the associated diseases.

## Background

Oral Submucous Fibrosis (OSMF) is a potentially malignant disorder, predominantly found in the oral cavity [1]. Arecoline, an enzyme found in the areca nut is found to be the commonest cause of oral submucous fibrosis [2]. South and Southeast asian countries have high prevalence of Oral Submucous Fibrosis due to the constant usage of tobacco and areca nut. Apart from OSMF, patients report to a dental hospital with other dental and systemic problems and are incidentally diagnosed with OSMF due to the lack of awareness of this deadly disease [3,4]. Schwartz first coined the term Oral Submucous Fibrosis after examining five women of Indian origin taken for a study in Kenya [5 - check with author]. Other names of OSMF include juxta-epithelial fibrosis, Idiopathic scleroderma of the mouth, Idiopathic palatal fibrosis etc. [6] This condition is initiated by the formation of vesicles and progress with the formation of fibrotic bands in the buccal and labial mucosa leading to limited mouth opening as stated by Pindborg in 1964 [7]. Oral submucous fibrosis, a generalized pathological condition of the oral mucosa is associated with the increased level of malignant transformation as per WHO [7]. Oral cancer has been found to be the fifth most common type of cancer in the world. Patients with OSMF are betel and pan chewers who develop the disease, which may progress to Oral cancer [8,9]. Etiology of oral submucous fibrosis is multifactorial. Areca nut chewing, tobacco usage, ingestion of chillies, genetic factors, and immunological reactions are known factors that pose risk in causing OSMF. [10,11] Clinically blanching and redness near the faucial pillars of the mouth are seen. As the severity of the disease increases, signs such as shrunken uvula, burning sensation, thick fibrotic bands on the buccal mucosa on palpation are confirmed which cause limited mouth opening [11]. Histo pathological features are characterised by juxta-epithelial inflammation with presence of oedema, fibroblasts and neutrophil and eosinophil inflammatory infiltrate. [12] Collagen bundles with uniform hyalinization and more chronic cell types such as plasma cells and B-lymphocytes are seen in later stages. Epithelial lining is thin (atrophic) and hyperkeratotic in nature [13]. There will be a decrease in tissue vascularity followed by muscle degeneration in successive stages of the disease [14]. This leads to irregular elastin deposits around the muscle fibres. Further progression causes difficulty in mouth opening due to the decreased destruction of type - 1 collagen and increased formation [15,16]. Treatment modalities in addition to surgical excision of fibrous bands include physiotherapy, chemopreventive agents such as green tea, retinoids and NSAID (Non steroidal anti inflammatory drugs) administration [17-19]. Polymorphism of various genes can cause increased susceptibility of OSMF and may cause malignant transformation [20-23]. The highest malignant transformation potential of OSMF compared to the other PMDs has also been the reason for the extensive research done on OSMF. Therefore the current survey was designed to assess the awareness about ORAL SUBMUCOUS FIBROSIS (OSMF) among patients, which is a deadly disease with a tortuous course.

## Methodology

A population of 100 patients who previously visited Saveetha Dental College and Hospital, under treatment for OMSF were considered for the study. The study was done under a University setting. Ethical approval was obtained from the Institutional Review Board (SDC/SIHEC/2020/DIASDATA/0619-0320). Among 86000 patients who had visited, between the time periods of June 2019 to March 2020 in the OPD of Saveetha Dental College and Hospital, 100 random patients diagnosed with OSMF were identified and included in the study. A questionnaire containing 20 questions was circulated online through Google forms among the selected patients. All the 20 questions were close ended. To eliminate bias a randomized sampling method was employed. The data was collected and tabulated in Google Excel Sheet, later fed into SPSS software for analysis. Dependent and independent variables were noted. Incomplete data regarding patient follow-ups and treatment completion were excluded from this survey. Chi square test was done to find the association between the various age groups and knowledge about various parameters in OSMF. (p value <0.05 was considered to be statistically significant.

## Results and Discussion:

Oral cancer has been found to be the fifth most common type of cancer in the world. Oral submucous fibrosis, a generalized pathological condition of the oral mucosa is associated with the increased level of malignant transformation according to WHO. Pathogenesis of oral submucous fibrosis is multifactorial inclusive of areca nut chewing, tobacco usage, and ingestion of chillies, genetic factors, and immunological reactions and sometimes known to be idiopathic [16]. In this study, 100 participants was the sample size. Out of which 9% of the participants were female patients and remaining 91% of them were male patients (Figure 1 -see PDF). Males were predominantly affected by OSMF compared to female patients in our study similar to previous studies. [6] Age distribution in our study shows us that the age group 20-30 years were less prone to OSMF compared to the age group 30-40 years, 40-50 years and above 50 years (Figure 2 - see PDF). High predilection was indicated in the middle age group of 30-40 and 40-50 years of age similar to the age group mentioned in previous literature [7] 93% of the surveyed population responded that they have been using tobacco products whereas the remaining 7% have OSMF symptoms which may be idiopathic or not caused by tobacco and areca nut usage (Figure 3 - see PDF). The frequency of response indicating tobacco chewing habits reveal that a majority of the population have the habit of chewing tobacco and areca nuts during the treatment period whereas the remaining population left the habit under the advice of a dentist or do not have any such habits comparable to previous literature [24]. Out of the 100 respondents, 61% of the population had burning sensation whereas 39% of the population did not report any signs of burning sensation (Figure 4 - see PDF). They also indicated to present with difficulty in opening their mouths and inability to move the tongue. This is due to associated inflammatory response, hyalinisation and loss of collagen bundles type 1 [16]. 81% of the patients were not aware of malignant transformation potential of OSMF and 19% of the population were aware (Figure 5 - see PDF). Many articles have suggested the increased malignant transformation potential of OSMF to OSCC due various to genetic and molecular factors [21,22]. Reduced awareness on the malignancy potential of OSMF may be due to lack of knowledge among the general population about this disease. Among the participants, when asked about the idealistic treatment modality for OSMF around 37% of the people considered surgery as the most idealistic treatment option. 14% of the population preferred supplements for treating this disease. 4% of the population considered physiotherapy and mouth opening exercises, 11% preferred hydrocortisone intramuscular injections and remaining 34% considered that all the treatment mentioned above should be taken simultaneously according to the stage of OSMF (Figure 6 - see PDF). There is an increase in the awareness of treatment preference among the population compared to the 28% of surgery preference and 21% of simultaneous treatment approach as per previous study indications [15,[17]. Association between the various age groups and their awareness on the various parameters was analysed using chi-square test. (Figure 7-15 - see PDF) 85% of the population are new to the term Oral Submucous Fibrosis. This is due to the lack of awareness and reduced level of knowledge on OSMF. Enormous research is being conducted on the appearance and correlation of OSMF with other systemic diseases, which is still questionable due to the intriguing course of the disease, which affects the entire human body [1]. Students can be trained to take histo pathological photomicrographs of patients with OSMF, correlate clinical features and offer a thorough description of the disease to the patients as a key to improve the awareness among the patients with or without the disease [25] Many research studies have been conducted to confirm oral squamous cell carcinoma in serum and saliva, similar studies are warranted in potentially malignant disorders like OSMF to hasten the process of diagnosis and treatment and hence reduce the progression to malignancy [20]. The limitations/ Cons of this study were limited geographic restrictions, lethargic attitude of patients, insufficient data with incomplete follow up. Futuristic scope of exploration in regards to OSMF with a more widely carried out studies in different regions of the South Asia to improve the knowledge among the diseases individuals.

## Conclusion

Data shows that 81% of the subjects were not aware of the malignancy caused by OSMF. A significant number of males use more tobacco than females (p value = 0.006 (<0.05) in the sample dataset. Thus, there is a need to create awareness on OSMF among the general population to combat the associated diseases.
